# Bio-Preservative Potential of Microorganisms Isolated from Red Grape against Food Contaminant Fungi

**DOI:** 10.3390/toxins13060412

**Published:** 2021-06-10

**Authors:** Victor Dopazo, Carlos Luz, Jordi Mañes, Juan Manuel Quiles, Raquel Carbonell, Jorge Calpe, Giuseppe Meca

**Affiliations:** Laboratory of Food Chemistry and Toxicology, Faculty of Pharmacy, University of Valencia, Av. Vicent Andrés Estellés s/n, 46100 Burjassot, Spain; victor.dopazo@uv.es (V.D.); jorge.manes@uv.es (J.M.); juan.quiles@uv.es (J.M.Q.); racar3@alumni.uv.es (R.C.); jorge.calpe@uv.es (J.C.); giuseppe.meca@uv.es (G.M.)

**Keywords:** bio-preservation, antifungal activity, grapes, metabolic profiling

## Abstract

Fungal spoilage is one of the main reasons of economic losses in the food industry, especially in the wine sector. Consequently, the search for safer and new preservation techniques has gained importance in recent years. The objective of this study was to investigate the antifungal and anti-mycotoxigenic activity from 28 microorganisms (MO) isolated from red grape. The antifungal activity of a cell free supernatant of fermented medium by the isolated MO (CFS) was tested with the agar diffusion method and the minimum inhibitory concentration (MIC) and minimum fungicidal concentration (MFC) assay. Additionally, different antifungal compounds from the CFS were identified and quantified (organic acids, phenolic compounds, and volatile organic compounds). Finally, the most active CFS were tested as red grape bio-preservative agents. Results evidenced that CFS fermented by the strain UTA 6 had the highest antifungal activity, above all isolates, and produced a wide pool of antifungal compounds. The use of UTA 6 CFS as bio-preservative agent showed a reduction of 0.4 and 0.6 log_10_ spores per gram of fruit in grapes contaminated by *A. flavus* and *B. cinerea*, respectively. Moreover, UTA 6 CFS treatment reduced the occurrence of aflatoxin B_1_ and fumonisin (B_2_, B_3_, and B_4_) production in grapes contaminated by 28–100%.

## 1. Introduction

Along the different stages of production, fruits and vegetables are susceptible to a wide variety of pathogens, such as nematodes, insects, and different microbial organisms [1]. Among those pathogenic agents, fungi represent a group that causes more damage to the agricultural sector [2]. Fungi alter food by decreasing the self-life and the appeal of the product and could produce a wide variety of toxigenic compounds such as mycotoxins [3,4]. 

Grape berries are one of those products primarily affected by fungal contamination, especially by the species of fungi belonging to the *Aspergillus*, *Botrytis*, *Fusarium*, and *Penicillium* genera [5]. The mycotoxins produced by them, including aflatoxins, ochratoxin A, fumonisins, zearalenone, trichothecens, penicillic acid, and patulin, are some of the most toxic secondary metabolites commonly found in food contaminated by fungi. Many of these mycotoxins are classified by the International Agency for Research on Cancer (IARC) as carcinogenic (aflatoxins), probably carcinogenic, and possibly carcinogenic (fumonisin B_1_, fumonisin B_2_, and ochratoxin A) to humans [6]. The use of synthetic compounds has been the regular measure against fungal contaminations. Nevertheless, the relation with different health and environmental problems and the rising concern of the consumer about those issues have increased the research interest to use and develop new and safer methods to manage fungal contamination [7]. 

New and safer techniques have been developed, such as the use of irradiation methods [8], thermal treatments [9], use of essential oils [10], biofilms, or the use of microbial antagonists [11]. Microbial antagonists, also known as bio-preservation agents, are a series of fungi, yeast, and bacteria which exhibit different antifungal activities [12]. The occurrence of antagonist microorganisms (MO) is a phenomenon which happens in nature as a result of the competition from the microbiome present in all foods [8]. Research and isolation of MO with antifungal properties has become a trend in the development of new methods to substitute the use of synthetic origin compounds [2]. In the bibliography, this antifungal activity has been reported with many MO, just as *Brevibacillus laterosporus* [13], *Trichoderma asperellum* [14], *Pseudomonas* sp. [15], and many acid lactic bacteria (LAB) like *Lactobacillus plantarum* [16,17], *Lactobacillus coryniformis* [18], or *Leuconostoc mesenteroides* [4]. 

The aim of this study was: (a) to evaluate the antifungal activity by quantitative and qualitative methods of a cell free Man Rogosa and Sharpe broth (MRS) fermented by MO isolated from red grape; (b) to identify and quantify the different components present in the fermented MRS; (c) to study the bio-preservative properties from the fermented MRS on red grape contaminated by fungi; and (d) to study the differential metabolomics profiling of fungal growth on contaminated grapes.

## 2. Results and Discussion

### 2.1. Isolation and Identification of LAB Strains

From the 33 MO isolated, a total of 28 were selected after the gram stain results. From the 28 isolates, the two MO with the highest activity against fungal contaminants of grape were selected and identified by identified by peptide mass fingerprinting MALDI-TOF MS. Identifications to the species level with a Log (score) superior to 2 were considered. Both isolates were classified to the species *Leuconostoc fallax*. The isolates were identified to the strain levels as *L. fallax* DSM 10614 (UTA 6) and *L. fallax* DSM 10615 (UTA 7). Moreover, the identity of the two strains was confirmed by the full sequence of the 16S rRNA obtained and compared to those already deposited in NBCI using BlastN tool.

Several studies report the presence of this bacterial species in different vegetal origin sources, like grapes [19], chickpeas, tapioca, and fermented derivates like wine [20] or sauerkraut [21], but only a few researches have reported the use of this species as bio-preservative agents. In Trias et al. [22] a cell free supernatant (CFS) made by the bacteria reduced the presence of *Listeria monocytogenes* in plants and vegetables. Nevertheless, this article is the first detailing the potential of this species as bio-protector agent of grapes.

### 2.2. Antifungal Activity Assays

The antifungal activity produced by the cell free supernatant from fermented MRS (CFS) obtained by the grape isolates was determined by the qualitative method agar diffusion test (Table 1). Thereby, 23 out of 28 the CFS manifested an inhibition halo against more than 80% of the fungi tested. Overall, the isolate UTA 6 manifested the greatest antifungal activity by showing inhibition halos against all 11 fungi. The highest inhibition halos were reported in the CFS from the strain UTA 2, which obtained inhibition halos more than 1 cm against *P. expansum* CECT 2278 and *P. commune* CECT 20767. The *Fusarium* spp., *P. expansum* CECT 2278, and *P. commune* CECT 20767 were the most sensitive fungi to the treatment. The strain *P. commune* CECT 20767 was inhibited by a 100% of the CFS used. Further, more than 85% of the CFS evidenced antifungal activity against the strains *F. verticillioides* ITEM 12043 and *F. graminearum* ITEM 126. On the contrary, *Aspergillus* genera evidenced the highest resistance against all CFS, especially the strain *A. carbonarius* ISPA 5010, which was only inhibited by five of the treatments tested.

The antifungal activity of the CFS from the grape isolated MO was carried out by MIC MFC assay (Table 2). The MIC values reported for *Penicillium* spp., *Aspergillus* spp., *Alternaria* spp., *Fusarium* spp., and *Botrytis cinerea* were in ranges from 12.5 to >100.0, 25.0 to >100.0, 6.3–25.0, 3.1–50.0, and 25.0–50.0 g/L, respectively. The MFC values differ from 12.5 to >100.0 g/L against *Penicillium* spp., 50.0 to >100.0 g/L for *Aspergillus* spp., 25.0–50.0 g/L for *Alternaria* spp., 6.3–100.0 g/L for *Fusarium* spp., and 50.0 to >100.0 g/L for *Botrytis cinerea*. The CFS had low activity against *Aspergillus* spp. and, as in the diffusion agar test, the strain *A. carbonarius* ISPA 5010 evidenced the highest resistance reaching MFC values superior to 100 g/L. On the contrary, the *Fusarium* and *Alternaria* spp. were the most sensitive to the treatment. The strains *Alternaria alternata* ITEM 8121, *Fusarium verticillioides* ITEM 12043 and *Fusarium proliferatum* ITEM 12072 presented the average lower values of MIC and MFC. With respect to the CFS, the isolated UTA 6, UTA 7, and UTA 9 showed antifungal activity against a greater fungal range. 

The CFS reported in this study exhibit similar results compared to other cell free supernatants made by different MO. The *Aspergillus* genera tends to be the most resistant to CFS fermented by MO. In opposition, *Fusarium* spp. and *Alternaria* spp. seem to be more susceptible to these treatments [11,23,24]. This is an interesting result because very few articles report the activity of a CFS against *Botrytis cinerea* [25].

Regarding the data obtained by the MIC MFC assay, alike values can also be seen in the literature using other CFS. Izzo et al. [26] reported on MFC values of CFS of sweet whey fermented by different strains of *L. plantarum* in a range of 31.0–250.0 g/L against *Aspergillus* spp. Or in Martí-Quijal et al. [27] where a fermented broth made out of by-products from the fish industry evidenced MFC of 16.0–31.0 g/L against a *Fusarium* spp. 

### 2.3. Identification and Quantification of Compounds Present in the CFS

The accumulation of organic acids, lactic acid, and acetic acid in the CFS is presented in Table 3a. Concentrations of lactic acid and acetic acid ranged from 1.5 to 4.5 g/L and 0.3 to 2.2 g/L, respectively. Lactic acid was found in higher concentrations in the CFS produced by the MO UTA 7, UTA 8, and UTA 15. No significant statistical differences were detected in the content of acetic acid (*p* < 0.05) quantified by liquid chromatography. The analysis of the phenolic compounds revealed the presence from one of the five phenolic compounds studied, phenyllactic acid (PLA) (Table 3b). Among the CFS in which the phenyllactic acid was detected, the concentrations ranged from 0.6 to 1.4 mg/L. This compound was more abundant in the CFS produced by UTA 3, UTA 6, and UTA 9. Previous studies report the activity of these three compounds as antimicrobial agents against a wide spectrum of MO contaminants, from yeast to molds [28,29]. Nevertheless, there is not unanimous opinion concerning the concentrations of these compounds required to inhibit fungal growth. Gerez et al. [30] report MIC values of lactic acid from 2.5 to 300.0 mM, of acetic acid from 0.3 to 120.0 mM, and phenyllactic acid 0.02–6.0 mM against *F. graminearum* and *A. niger*. Izzo et al. [26] also link higher productions of PLA in medium fermented by acid lactic bacteria with rising antifungal activities against *Aspergillus*, *Penicillium*, and *Fusarium* spp.

Between the volatile organic compounds (VOCs) studied a total of 36 compounds were detected and quantified in the CFS and the MRS control (Table S1). The VOCs were divided in four groups according to their chemical groups, they were classified as alcohols, aldehydes, pyrazines and others. Compared to the samples, the MRS control evidenced a tiny spectrum of different compounds and the more abundant chemicals were 2,4-Di-tert-butylphenol and the aldehydes. Fermentation of the MRS by the isolates showed an increase on the pool of compounds present in the CFS, such as ethanol, pyrazines, and acetic acid. In particular, the concentration of different pyrazines significantly increased between 10% and 40%. The antifungal activity of pyrazines is well known in the literature. Many articles report the effect of this volatile compound on the inhibition of different *Penicillium* and *Fusarium* [31,32].

### 2.4. Leuconostoc fallax DSM 10614 and DSM 10615 as a Grape Bio-Preservative

**Figure 1:**
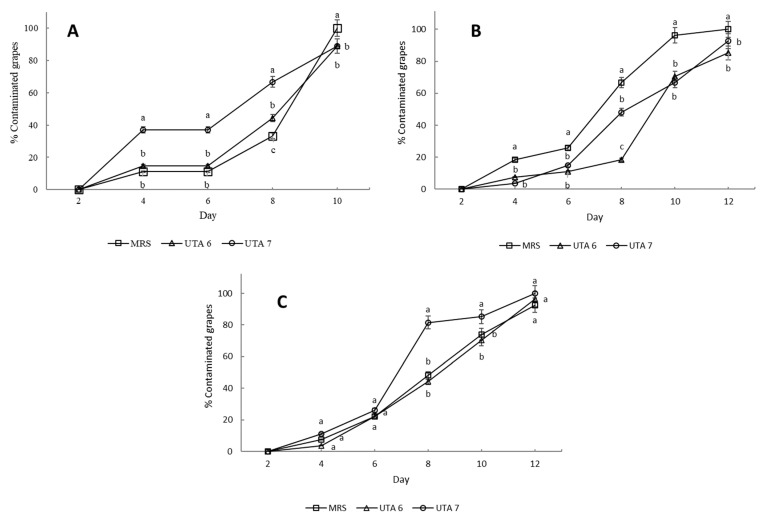
Results of the bio-preservation test on red grape contaminated with *B. cinerea* (**A**), *Aspergillus flavus* (**B**), *Aspergillus niger* (**C**). Treatment used were MRS as control, and CFS fermented by the strains UTA 6 and UTA 7. Values were expressed as mean ± standard deviations of triplicates. Statistically significant differences between the mean of contaminated percentage grapes during storage time and the type of treatment are indicated with different letters, (*p* < 0.05).

**Figure 2:**
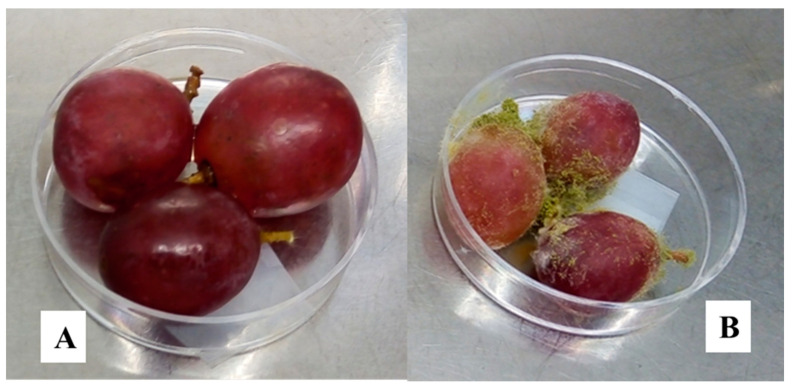
Fungal growth of *A. flavus* on grapes after 12 days at room temperature and in the dark conditions. A negative growth (**A**) and a positive growth (**B**).

**Figure 3:**
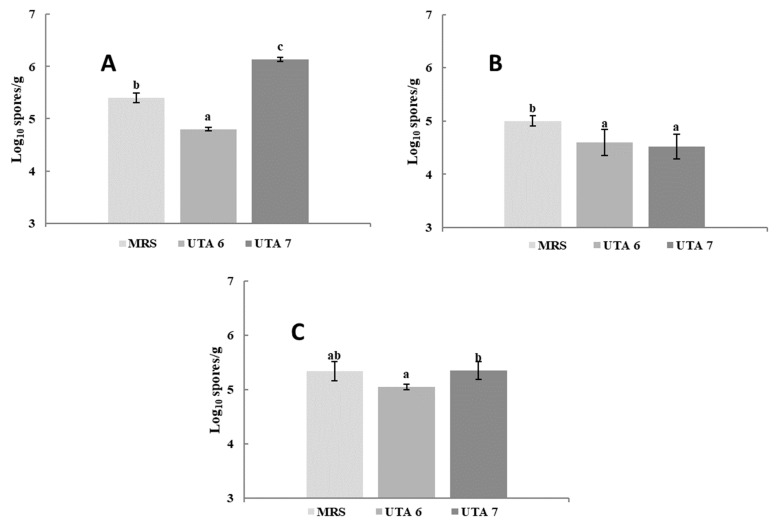
Results of the determination of the fungal population in red grape contaminated with *B. cinerea* (**A**), *Aspergillus flavus* (**B**), *Aspergillus niger* (**C**). Treatment used were MRS as control, and CFS fermented by the strains UTA 6 and UTA 7. Values were expressed as mean ± standard deviations of triplicates. Statistically significant differences between the mean content of spores/g and the type of treatment are indicated with different letters, (*p* < 0.05).

The bio-preservative effect of the CFS fermented by *L. fallax* DSM 10614 (UTA 6) and DSM 10615 (UTA 7) of red grapes inoculated with *A. flavus*, *A. niger* and *B. cinerea* during incubation can be seen in Figure 1. Fungal growth determination was performed as seen in Figure 2. After 12 days of incubation, grapes treated with CFS fermented by UTA 6 evidenced a significant reduction of 15% of the infected grapes inoculated with *A. flavus* (*p* < 0.05) compared to the MRS control. Moreover, in the control grapes contaminated with *B. cinerea* reached a contamination of 100% after 10 days, although when the CFS fermented by UTA 6 was applicated, contamination was reduced to 88%. No visible augment of the shelf life of the fruit was achieved in the grapes inoculated with *A. niger*. The results of the microbiological analysis from the grapes confirmed the results evidenced by the study of the self-life (Figure 3). After 12 days, the control grapes showed a population of 5.00 log_10_ spores/g, whereas the fungal population was reduced significantly to 4.60 and 4.50 log_10_ spores per gram of fruit (*p* < 0.05) in grapes treated with the CFS fermented by UTA 6 and UTA 7, respectively. In grapes inoculated with *B. cinerea,* the fungal population in control grapes reached 5.40 log_10_ spores/g, while in the grapes treated with CFS produced by UTA 6, a significant decrease of the fungal growth was achieved, 4.80 log_10_ spores/g (*p* < 0.05). However, the CFS fermented by UTA 7 showed a significant increase in the number of *Botrytis cinera* spores per g of grape compared to the control and the CFS by UTA 6 at the end of the storage period. The concentration of secondary metabolites derived from fermentation in the food assay was not sufficient to inhibit fungal growth, increasing the spore formation.

There are no previous publications in the bibliography related to the use of CFS produced by LAB strains for the bio-preservation of red grapes. Nevertheless, different studies have proven the bio-preservation of red grape with other methods. For instance, Lappa et al. [33] evidenced the antifungal activity of a *Lactobacillus plantarum* strain against *Aspergillus* spp. The immersion of the grapes in a suspension of 10^8^ CFU/mL of the LAB studied was able to delay the fungal growth two days. Moreover, other bacterial species have been used as bio-preservatives, such as in the work of Kasfi et al. [34], where strains of *Bacillus* spp. reduced the growth of *A. flavus* a 27% in grapes treated with a suspension (10^7^ CFU/mL) of the antagonistic MO. Previous studies reported bio-preservative effects of CFS fermented by *L. plantarum* TR7 and TR71 against *Aspergillus flavus* ITEM 8111 and *Penicillium expansum* CECT 2278 on tomato [12].

### 2.5. Metabolite Profiling of Fungal Growth on Grape

**Figure 4:**
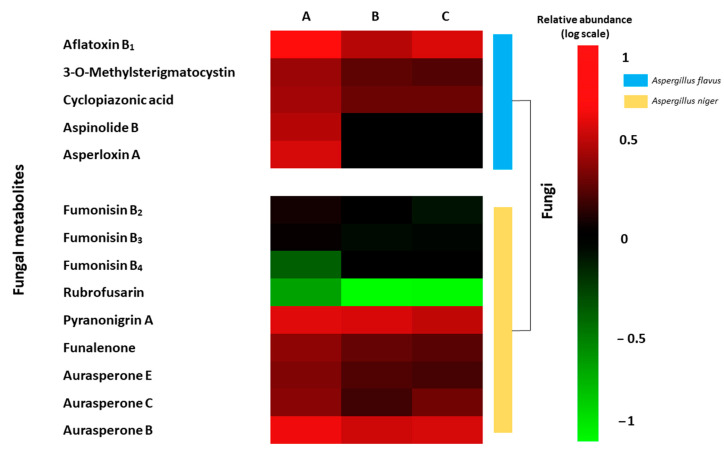
Heat map of the fungal metabolites produced by *Aspergillus flavus* and *Aspergillus niger* on inoculated grapes treated ((**A**) Control; (**B**) UTA 6; (**C**) UTA 7) after 12 days of incubation at room temperature. Colors are based on relative abundance (logarithmic scale) of metabolites produced independently for each fungus, where red represents high abundance and green represents low abundance.

The LC-MS-TOF-based metabolomics approach is an interesting tool to study changes in the fungal metabolomics profiling. In the present study, the differential metabolites profile expressed by *A. flavus* and *A. niger* on inoculated grapes and treated by CFS were evaluated. A total of 14 toxic metabolites were identified. *A. flavus* produced on inoculated grapes a differential production of aflatoxin B_1_, 3-O-methylsterigmatocystin, cyclopiazonic acid, aspinolide B, and asperloxin A. However, *A. niger* showed the production of other fungal metabolites like fumonisin B_2_, B_3_, B_4_, rubrofusarin, pyranonigrin A, funalenone, and aurasperone E, C, and B. In Figure 4 shows the heat map of the differential metabolite profiling produced by *A. flavus* and *A. niger* between the three grapes treatments. Differences were also shown in the abundance of fungal metabolites between the two bio-preservation treatments using CFS fermented by UTA 6 and UTA 7. Similar activities have been evidenced in the bibliography with other bio-preservative compounds. In the work of Císarová et al. [35], the use of different essential oils as bread preserving agents also evidenced an influence on the metabolite production of several *Aspergillus* species, reducing the occurrence of mycotoxins compared to the control.

**Figure 5:**
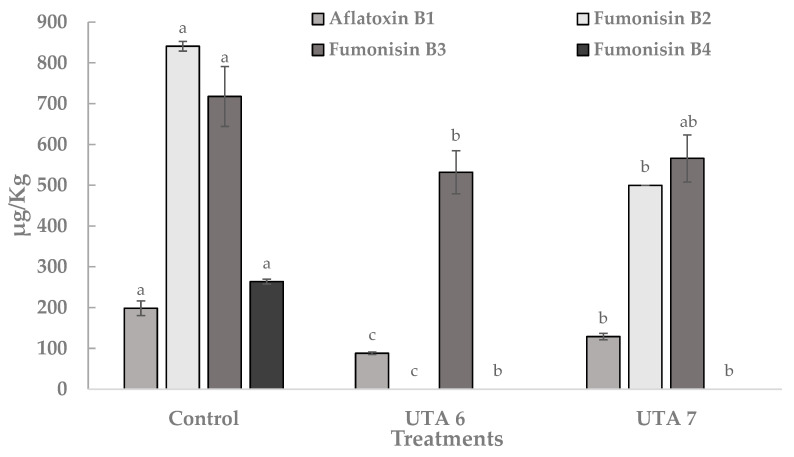
Quantification of mycotoxin aflatoxin B_1_, fumonisin B_2_, fumonisin B_3_, fumonisin B_4_ produced by *Aspergillus flavus* and *Aspergillus niger* on inoculated grapes treated after 12 days of incubation at room temperature. Values were expressed as mean ± standard deviations of triplicates. Statistically significant differences between the mean content of each mycotoxin and the type of treatment are indicated with different letters, (*p* < 0.01).

Frequently monitored mycotoxins in food like aflatoxin B_1_, fumonisin B_2_, fumonisin B_3_, fumonisin B_4_ produced by *Aspergillus flavus* and *Aspergillus niger* were quantified using calibration curves with commercial standards. In Figure 5, the content of these mycotoxins expressed in µg/Kg is reported. In the control grapes, the content of aflatoxin B_1_, fumonisin B_2_, fumonisin B_3_ and fumonisin B_4_ were 198 µg/Kg, 841 µg/Kg, 718 µg/Kg, and 264 µg/Kg. Bio-preservation of grapes using CFS evidenced a statistically significant reduction in the mean content of the four mycotoxins. Moreover, the fumonisin B_2_ and fumonisin B_4_ was not detected on treated grapes using CFS fermented by UTA 6. In general, the UTA 6 treatment showed significant reductions in the content of the 4 mycotoxins compared to the UTA 7 treatment. Other sources have report this anti-mycotoxigenic activity from other LAB strains. In the work of Nazareth et al. [36], the production of several metabolites in a CFS fermented by *Lactobacillus plantarum* CECT 749 has been linked to a reduction of aflatoxin B_1_ and fumonisin B_1_ in corn contaminated by *A. flavus* and *F. verticillioides*. Guimarães et al. [37] also reported anti-aflatoxigenic activity in vitro related with compounds such as lactic acid, PLA, hydroxyphenyllactic acid, and indol acetic acid present in CFS fermented by *L. plantarum* UM55 against *Aspergillus parasiticus* and *A. flavus*. They reported a percentage reduction in aflatoxins production in the range of 53–97%.

## 3. Conclusions

The in vitro experiments demonstrated that CFS produced by UTA 6 has a significant antifungal activity against a wide variety of different fungal species. Furthermore, this antifungal activity may be related to the production of a biocomplex of antimicrobial compounds, including lactic acid, acetic acid, PLA, and pyrazines. The application of this CFS evidenced a reduction in the fungal growth in grapes contaminated with *A. flavus* and *B. cinerea*. The application of the UTA 6 CFS managed to significantly reduce the occurrence of aflatoxin B_1_, fumonisin B_2_, fumonisin B_3_, and fumonisin B_4_ in the grapes contaminated by *A. flavus* and *A. niger*. Therefore, applications of this method may present a promising increase in the self-life of grapes as a post-harvest treatment, reduce the use of traditional antifungal compounds, and have a significant impact on human health, reducing the presence of toxic metabolites produced by the species tested. Further investigations will focus on the pre-harvest application of CFS in the field to prevent fungal development and reduction of mycotoxin production in grapes.

## 4. Materials and Methods

### 4.1. Chemicals and Materials

The media cultures used for the study were Man Rogosa and Sharpe broth (MRS). Man Rogosa and Sharpe agar (MRS-A), potato dextrose broth (PDB), and potato dextrose agar (PDA) were all purchased from Liofilchem (Teramo, Italy). The deionized water (<18 MΩ/cm) was obtained from a Milli-Q water purification system (Millipore Corp., Bedford, MA, USA). 

Lactic acid was from Sigma-Aldrich (St. Louis, MO, USA) and acetic acid were obtained from Fisher Scientific (Waltham, MA, USA). Gallic acid, chlorogenic acid, caffeic acid, syringic acid, vanillic acid, p-coumaric, hydroxybenzoic acid, vanillin, hydroxycinnamic acid, sinapic acid, benzoic acid, phenyllactic acid, dihydrocaffeic acid, 3,4-dihydroxyhydrocinnamic acid, and DL-p-hydroxyphenyllactic acid were acquired from Sigma–Aldrich (Dublin, Ireland). Phenyllactic acid was obtained from BaChem (Weil am Rhein, Germany). Ferulic acid was purchased from MP Biomedicals, and protocatechuic acid came from HWI Pharma Services (Ruelzheim, Germany). All analytes had a purity of 95%.

Mycotoxins aflatoxin B_1_ and fumonisin B_1_ were provided from Sigma–Aldrich (St. Louis, MI, USA) with a purity ≥95%.

The filamentous fungi used in this study were *Penicillium commune* CECT 20767, *Aspergillus niger* CECT 2088, *Botrytis cinerea* CECT 20973, *Penicillium expansum* CECT 2278, and *Penicillium digitatum* CECT 2954 from the Colección Española de Cultivos Tipo (CECT) (Valencia, Spain) and *Aspergillus carbonarius* ITEM 5010, *Fusarium verticillioides* ITEM 12043, *Fusarium proliferatum* ITEM 12072, *Fusarium graminearum* ITEM 126, *Aspergillus flavus* ITEM 8111, *Alternaria alternata* ITEM 8121, purchased from the ITEM Collection from Istituto di Scienze delle Produzioni Alimentari (ISPA) (Bari, Italy). 

### 4.2. Microorganisms Isolation

The MO investigated in this study were isolated from red grapes from different Tempranillo wine grapes vineyards located in Villar del Arzobispo (Valencia, Spain). Grapes were homogenized in distilled water with 0.1 % peptone water using a using a Stomacher (IUL, Barcelona, Spain). The homogenized mix was used to inoculate MRS. After 24 h at 37 °C, serial dilutions of the incubated MRS were sowed in MRS agar. After an incubation of 24 h at 37 °C different colonies were taken and cultured on new MRS agar plates by the streak plate technique [38]. This incubation was performed also under anaerobic conditions. After isolation, the MO were subjected to a Gram staining and a morphological analysis by microcopy. Gram positive bacteria and yeast were selected for the study. Selected MO were frozen with MRS broth, with 25 % of glycerol, at −80 °C. For the preparation of the treatments studied in this work, the MO were recovered in MRS broth medium 37 °C for 24 h. 

### 4.3. Use of a MALDI-TOF MS System and 16S rRNA Gene Sequencing for Bacterial Identification

Identification of the strains with the highest antifungal activity was performed as described in [39]. The analysis was carried out from an isolated culture of the bacteria. The equipment used was a MALDI-TOF MS with a mass spectrophotometer Microflex L20 (Bruker Daltonics) and a N” laser. The acquisition of all spectrums was in positive linear ion mode. The voltage acceleration was 20 kV and the mass range was set from 2000–20,000 Da. Following the method MALDI Biotyper Realtime Classification (RTC), three spectrums were obtained from each sample. The identification corresponded to the highest log score. The results were compared with the database MBT 7854 y MBT 7311_RUO (Bruker Daltonics). Moreover, 16S rRNA gene sequencing of isolates was performed using the method described by Chenoll et al. [40].

### 4.4. Preparation of the CFS

The CFS was made following the next steps. After their defrost and recovery, the MO were cultivated in MRS at 37 °C until the exponential phase growth (12 h). Then, MRS was inoculated with MO in a 1/100 (*v*/*v*) proportion and incubated for 72 h at 37 °C. Afterwards, a CFS was obtained by centrifugation at 4000 RPM for 15 min at 4 °C and biomass was discarded. The CFS was frozen at −20 °C, then lyophilized (FreeZone 2.5 L, Labconco, Kansas City, MO, USA) to be used in subsequent assays. 

### 4.5. Antifungal Activity Assays by Qualitative Method

Agar diffusion method was performed as described in Luz et al. [12]. A total of 11 fungal strains were cultured on PDA plates using sterile cotton swabs. Then wells were punched with sterile 1000 µL pipette tips. Finally, 100 µL from a 400 g/L suspension of each CFS was added to each well and the plates were incubated at 25 °C for 48 h under aerobic conditions. Each experiment was carried out in triplicate and the average of the three measured diameters was used. Antifungal activities were recorded as “++” when the inhibition halo reached more than 1 cm of diameter, as “+” when the inhibition halo was inferior to 1 cm, or “-” when no inhibition halo was observed. MRS suspended at 400 g/L was used as control. 

### 4.6. Antifungal Activity Assays by Quantitative Method

The technique used for this analysis was the minimum inhibitory concentration (MIC) and minimum fungicidal concentration (MFC) method as described by Nazareth et al. [36]. This test was performed using the CFS that evidenced the greatest results in the quantitative test. Trials were performed per quadruplicate. For each of them, a positive control, which consisted in 100 µL of sterile PDB inoculated with 100 µL of the spore suspension (10^5^ spores/mL), and a negative control (200 µL sterile PDB) was performed. In a 96-well plate, 100 µL of a solution of PDB with 10^5^ spores/mL was mixed at a 1:1 (*v*/*v*) ratio with different concentrations, from 200.0 to 0.1 g/L, of the CFS. Then, after 72 h of incubation at 25 °C, the MIC was identified as the smallest concentration in which there was an absence of fungal growth compared to the positive control.

Afterwards, 10 µL from the MIC well and higher concentrations were plated on PDA and incubated for 72 h at 25 °C to control the growth of the species tested. Then, the MFC was described as the lowest concentration in which no fungal growth was observed. 

### 4.7. Determination of Organic Acids and Phenolic Compounds in the CFS

The organic acids (lactic acid and acetic acid) were analysed using a JASCO Analytica (28600 Mary's Ct, Easton, MD, USA) high-performance liquid chromatography (HPLC) system equipped with a quaternary pump, an MD 4015 PDA diode array detector, a 20 µL sample injection loop, and a Rezex ROA-Organic Acid (150 × 7.8 mm) reverse phase column (Phenomenex Inc. 411 Madrid Avenue, Torrance, CA, USA). Mobile phase consisted in an isocratic solution of water and formic acid at 0.1% (*v*/*v*) flowing at a rate of 0.8 mL/min for 20 min. Assay was performed with wavelength of 214 nm for quantification [41]. Calibration curves were performed using lactic acid and acetic acid in MRS Broth, diluted 1/20 (*v*/*v*), at a standard final concentration from 0.125 to 1 g/L. A total of 3 replicates were performed of each CFS (independent fermentations) and the results were given in g/L.

For the analysis of the phenolic compounds, fist a QuEChERS extraction of the CFS was performed [42]. A total of 10 mL of the samples was mixed by vortex with a solution of 4 g de MgSO_4_, 1 g NaCl, and 10 mL of ethyl acetate (with 1% of formic acid). After centrifugation, the supernatant was added to 150 mg C18 and 900 mg MgSO_4_ and vortexed for 1 min. Then samples were centrifuged again, and the supernatant was dried under nitrogen flow until the analysis.

Before the injection in the chromatograph samples were suspended in 1 mL with a solution of water with acetonitrile 10% (*v*/*v*). The chromatograph used was an Agilent 1200 (Palo Alto, CA, USA) equipped with a vacuum degasser, a binary pump and autosampler. A Gemini C18 column (50 9 2 mm, 100 Å and particle size 3 μm; Phenomenex) was used as stationary phase and 0.1% FA in water (solvent A) and 0.1% FA in ACN (solvent B) as the mobile phase. The elution rate used was: 0 min, 5% B; 30 min, 95% B; 35 min, 5% B. The volume used for each injection was 20 µL. The flow rate was set at 0.3 mL/min and a run time of 37 min. 

The mass spectrophotometry (MS) study a was executed with a Q-TOF-MS (6540 Agilent Ultra High-Definition Accurate Mass), equipped with an Agilent Dual Jet Stream electrospray ionisation (Dual AJS ESI) operating in the negative ion mode. Parameters were set at; capillary voltage 3.5 kV, drying gas flow (N_2_) 8.0 L/min, nebuliser pressure 30 psig, temperature of 350 °C and fragmentor voltage of 175 V. Targeted MS/MS study was performed using collision energy of 10, 20 and 40 eV. Calibration curves with all phenolic compound standards described in the Section 4.1 were carried out using a concentration range of 0.01 to 1 mg/L. Software use for the data integration was MassHunter Qualitative Analysis Software B.08.00 [43]. A triplicate of analysis was performed for each sample.

### 4.8. Determination of Volatile Organic Compounds (VOC) in the CFS

The analysis of the VOC present in the CFS was performed using a headspace solid-phase microextraction (SPME) and a single quadrupole mass spectrometer detector (GC/MS) Agilent 6890N GC system (Agilent Technologies, Palo Alto, CA, USA). A quantity of 200 µg of the CFS was diluted in 2 mL of water and incubated at 55 °C for 45 min. Then, the VOC were extracted of the headspace using a SPME (Supelco, Bellafonte, PA, USA) with a fiber coated with a 50/30 μm layer of divinylbenzene/carboxen/polydimethylsiloxane (DVB/CAR/PDMS). Volatiles were desorbed from the DVB/CAR/PDMS fiber 250 °C for 5 min inside the injector from the GC/MS in splitless mode. Helium (99.999%) was used as carrier gas set at a flow rate of 1.0 mL/min. Column used in the assay was an HP-5MS (30 m × 0.25 mm, 0.25 μm 5% diphenyl/ 95% dimethylpolysiloxane) capillary column (J&W Scientific, Folsom, CA, USA), and was set at a temperature of 40 °C, for 2 min, increased to 160 °C at 6 °C per min, and to ramp to 260 °C at 10 °C per min (held for 4 min). The ion set temperature was set at 230 °C, a mass range of 40–450 Da and an electron ionization energy of 70 eV. Identification of the compounds was performed using NIST Atomic Spectra Database version 1.6 (Gaithersburg, MD, USA) setting the similarity spectra at 95% [44]. A total of 3 replicates were performed of each CFS (independent fermentations). Results were expressed as mean (n = 3) ± standard deviation of percentage of each area by the total area of the chromatogram peaks. 

### 4.9. Protection of Red Grapes from Fungal Spoilage by CFS

Adapting the steps from Luz et al. the assay was performed as it follows [12]. Under sterile conditions in a Telstar MH 100 laminar flow hood (Terrassa, Spain) red grapes (from ESTABLECIMIENTOS MAS Y MAS S.L., Burjassot, Valencia, Spain), were decontaminated by submerging in a solution of water and sodium hypochlorite at 1%, then washed with sterile distilled water. A total of 81 grapes divided into 9 for each treatment and fungus were placed in groups of 3 in different petri dishes as replicates (n = 9). Each grape was punched with a sterile needle, then sprayed with 1 mL of a conidia solution (1.5 × 10^3^ spores/g). Afterwards, grapes were sprayed with 2 mL a solution of 500 g/L of the CFS reaching a final concentration of 50 g of CFS per Kg of fruit. Between each steep the grapes dried for 30 min. Then, the fruits were stored in a closed sterile plastic box (dimensions 30 cm × 40 cm) for 10–12 days at room temperature and in a dark condition. Finally, six replicates were homogenized at random, in a ratio of 1/10 (*w*/*v*) with distilled water, 0.1% of peptone (*w*/*v*), and 0.1% of Tween 80 (*v*/*v*), and three replicates of a serial dilution were performed and cultured on PDA plates. After 48 h at 25 °C, the CFU were observed. Results were reported in percentage of contaminated grapes per day and spores per gram of fruit.

### 4.10. Metabolite Profiling of Fungal Growth on Grape

To investigate the effects of different bio-preservative treatments on inoculated grape on the metabolite profiling of *A. flavus* ITEM 8111 and *A. niger* CECT 2088, we collected contaminated grapes at the end of the incubation period, following the method described by Quiles et al. with some modifications [45]. The samples were frozen at −80 °C and lyophilized for 2 days. After, the grapes were crushed using a Cecotec electric coffee grinder (Valencia, Spain) to reduce particle size. Then, 5 g of sample were extracted with 25 mL of methanol for 2 min using a homogenizer T 50 Ultra-turrax (Staufen, Germany). The supernatant was dried under nitrogen flow with a Buchi R-215 Rotavapor system (Essen, Germany). The dry samples were dissolved with 2 mL of methanol, filtered with 0.22 µm and analysed using an UPLC (1290 Infinity LC, Agilent Technologies) coupled with a quadrupole time of flight mass spectrometer (Agilent 6546 LC/Q-TOF) operating in positive ionization mode. Chromatographic separation was performed with an Agilent Zorbax RRHD SB-C18, 2.1 × 50mm, 1.8 µm column. Mobile phase A was composed of Milli-Q water and acetonitrile was used for mobile phase B (both phases were acidified with 0.1% formic acid), with gradient elution, as follows: 0 min, 2% B; 22 min 95% B; 25 min, 5% B. The column was equilibrated for 3 min before every analysis. The flow rate was 0.4 mL/min, and 5 μL of sample was injected. Dual AJS ESI source conditions were as follows: gas temperature: 325 °C; gas flow: 10 L/min; nebulizer pressure: 40 psig; sheath gas temperature: 295 °C; sheath gas flow: 12 L/min; capillary voltage: 4000 V; nozzle voltage: 500 V; Fragmentor: 120 V; skimmer: 70 V; product ion scan range: 100–1500 Da; MS scan rate: 5 spectra/s; MS/MS scan rate: 3 spectra/s; maximum precursors per cycle: 2; and collision energy: 10, 20, 40 eV. The analysis of the metabolites was carried out in triplicate. Calibration curves of aflatoxin B_1_ and fumonisin B_1_ at a concentration of 0.01 to 5 mg/L were performed to quantify the most important toxic secondary metabolites produced by *A. flavus* and *A. niger* in treated grapes. Fumonisins (B_2_, B_3_ and B_4_) were quantified using the fumonisin B_1_ calibration curve as a reference. Untargeted LC/Q-TOF based metabolomics approach were used identify the differential metabolic profiling of fungi growing on grapes treated with CFS. Integration, data elaboration, and identification of metabolites were managed using MassHunter Qualitative Analysis software B.08.00 and library PCDL Manager B.08.00.

### 4.11. Statistical Analysis

The statistical analysis was performed using the software InfoStat 2019 (Universidad Nacional de Córdoba, Córdoba, Argentina). The differences between the groups were analysed with one-way ANOVA followed by the Tukey HSD post-hoc test for multiple comparisons. The differences have been considered statistically significant at *p* < 0.05 and *p* < 0.01.

## Figures and Tables

**Table 1:** Antifungal activity of isolated microorganisms against *Penicillium*, *Aspergillus*, *Fusarium*, *Botrytis* and *Alternaria* species by agar diffusion method. Inhibition halos were marked as “++” when the halo reached more than 1 cm of diameter, as “+” when the inhibition halo was inferior to 1 cm and “-” when no inhibition halo was observed. The concentration of CFS used was 400 g/L. MRS medium was used as control.

**Fungi**	**Sample**														
	**MRS**	**UTA1**	**UTA2**	**UTA3**	**UTA4**	**UTA5**	**UTA6**	**UTA7**	**UTA8**	**UTA9**	**UTA10**	**UTA11**	**UTA12**	**UTA13**	**UTA14**
*P. expansum* CECT 2278	-	+	++	+	++	+	+	+	++	+	+	+	+	+	+
*P. digitatum* CECT 2954	-	-	-	-	+	-	+	-	-	+	-	+	+	+	-
*P. commune* CECT 20767	-	+	++	+	+	+	+	+	+	+	+	+	+	+	+
*A. flavus* ITEM 8111	-	-	+	+	+	+	+	+	+	+	+	+	+	+	+
*A. carbonarius* ISPA 5010	-	-	-	+	+	-	+	-	-	+	+	-	-	-	-
*A. niger* CECT 2088	-	+	+	+	-	+	+	-	+	-	+	+	-	+	+
*Al. alternata* ITEM 8121	-	-	+	+	+	+	+	+	+	+	+	+	+	+	+
*F. graminearum* ITEM 126	-	+	+	+	+	+	+	+	++	+	+	+	+	+	+
*F. proliferatum* ITEM 12072	-	-	+	+	+	+	+	+	+	+	+	+	+	+	+
*F. verticillioides* ITEM 12043	-	-	+	+	+	+	+	+	+	+	+	+	+	+	+
*B. cinerea* CECT 20973	-	-	+	+	+	+	+	+	+	+	+	+	+	+	+
**Fungi**	**Sample**														
	**UTA15**	**UTA16**	**UTA17**	**UTA18**	**UTA19**	**UTA20**	**UB5**	**UB6**	**UB7**	**UB8**	**UBC1**	**UTC6**	**UT5**	**VBC9**	
*P. expansum* CECT 2278	+	+	++	+	+	+	-	-	+	+	-	-	-	+	
*P. digitatum* CECT 2954	-	-	-	-	+	+	-	-	-	-	-	-	-	-	
*P. commune* CECT 20767	++	+	+	+	++	+	+	+	+	+	+	+	+	+	
*A. flavus* ITEM 8111	+	+	+	+	+	+	-	-	+	+	-	-	-	-	
*A. carbonarius* ISPA 5010	-	-	-	-	-	-	-	-	-	-	-	-	-	-	
*A. niger* CECT 2088	+	+	-	+	+	+	+	+	+	+	+	+	+	+	
*Al. alternata* ITEM 8121	+	+	+	+	+	+	-	-	+	+	-	-	-	+	
*F. graminearum* ITEM 126	+	+	+	+	+	+	-	-	+	+	+	-	-	+	
*F. proliferatum* ITEM 12072	+	+	+	+	+	+	-	-	+	+	-	-	-	+	
*F. verticillioides* ITEM 12043	+	+	+	+	+	+	-	-	+	+	+	+	+	+	
*B. cinerea* CECT 20973	+	+	+	+	+	+	-	-	+	+	-	-	-	+	

**Table 2:** Results of the Minimum Inhibitory Concentration (MIC) and Minimum Fungicidal Concentration (MFC) for lyophilised CFS expressed in g/L. nd = not detected.

Fungi	Samples													
	UTA2		UTA3		UTA4		UTA5		UTA6		UTA7		UTA8	
	MIC	MFC	MIC	MFC	MIC	MFC	MIC	MFC	MIC	MFC	MIC	MFC	MIC	MFC
*P. expansum* CECT 2278	13	13	13	13	13	13	13	13	13	25	25	25	13	25
*P. digitatum* CECT 2954	100	>100	50	>100	25	>100	25	>100	50	>100	100	>100	13	>100
*P. commune* ECT 20767	25	50	25	50	25	25	25	50	25	50	25	50	25	50
*A. flavus* ITEM 8111	50	50	50	100	50	50	25	50	25	50	25	50	50	50
*A. carbonarius* ISPA 5010	50	>100	50	>100	50	>100	50	>100	50	>100	50	>100	50	>100
*A. niger* CECT 2088	100	100	100	100	nd	nd	100	100	25	100	25	100	50	>100
*Al. alternata* ITEM 8121	13	25	13	25	13	13	13	50	6	25	13	25	13	25
*F. graminearum* ITEM 126	13	25	13	25	13	50	13	13	6	6	13	13	6	25
*F. proliferatum* ITEM 12072	13	25	13	25	13	25	13	50	13	13	13	50	13	50
*F. verticillioides* ITEM 12043	6	13	6	13	3	13	3	13	6	13	6	25	6	25
*B. cinerea* CECT 20973	50	100	50	>100	50	50	50	100	25	50	25	50	50	100
**Fungi**	**UTA9**		**UTA10**		**UTA11**		**UTA12**		**UTA13**		**UTA14**		**UTA15**	
*P. expansum* CECT 2278	13	50	25	50	25	>100	13	13	25	25	13	25	13	50
*P. digitatum* CECT 2954	25	>100	25	>100	25	>100	25	>100	100	>100	25	>100	50	>100
*P. commune* ECT 20767	25	50	25	50	25	50	25	50	50	50	25	50	25	50
*A. flavus* ITEM 8111	25	50	50	50	50	100	50	50	50	100	50	50	50	50
*A. carbonarius* ISPA 5010	50	100	50	100	50	>100	50	100	50	100	50	>100	50	100
*A. niger* CECT 2088	nd	nd	50	>100	50	>100	nd	nd	100	>100	100	100	100	>100
*Al. alternata* ITEM 8121	13	25	13	25	13	50	13	25	13	25	13	25	13	25
*F. graminearum* ITEM 126	6	50	6	50	6	50	13	25	13	25	6	50	6	50
*F. proliferatum* ITEM 12072	13	50	13	50	13	50	13	50	13	50	13	50	6	25
*F. verticillioides* ITEM 12043	3	13	3	6	3	6	13	13	3	25	3	25	3	13
*B. cinerea* CECT 20973	50	50	50	100	50	100	50	50	50	>100	50	>100	50	100
**Fungi**	**UTA16**		**UTA17**		**UTA18**		**UTA19**		**UTA20**		**UB7**		**UB8**	
*P. expansum* CECT 2278	13	25	13	50	13	50	13	50	13	50	13	25	13	22
*P. digitatum* CECT 2954	50	>100	50	>100	100	>100	100	>100	50	>100	50	>100	25	>100
*P. commune* ECT 20767	25	25	25	50	50	50	50	>100	50	50	50	50	25	50
*A. flavus* ITEM 8111	50	100	50	50	25	100	25	100	25	100	50	>100	50	100
*A. carbonarius* ISPA 5010	50	100	50	100	50	>100	50	100	50	100	100	100	100	100
*A. niger* CECT 2088	nd	nd	nd	nd	50	100	100	>100	100	>100	100	>100	100	>100
*Al. alternata* ITEM 8121	6	25	6	25	6	50	13	25	25	50	13	25	13	25
*F. graminearum* ITEM 126	13	25	13	50	13	50	13	100	6	100	6	13	25	25
*F. proliferatum* ITEM 12072	13	25	13	25	13	25	6	25	13	25	13	25	13	25
*F. verticillioides* ITEM 12043	3	13	3	13	3	6	3	6	3	13	6	13	6	6
*B. cinerea* CECT 20973	50	50	50	>100	50	>100	50	>100	100	>100	50	>100	50	100

**Table 3:** Identification and quantification of the (a) organic acids (g/L) and (b) phenolic compounds (mg/L) produced by the microorganisms isolated from grape in CFS. The results are expressed as mean ± standard deviation. Statistically significant differences for each fermentation using one-way ANOVA Tukey HSD post hoc test are indicated with different letters (*p* < 0.05).

**(a) Oraganic acids**	**UTA1**	**UTA2**	**UTA3**	**UTA4**	**UTA5**	**UTA6**	**UTA7**	**UTA8**	**UTA9**	**UTA10**
Lactic acid	1.5 ± 0.6 ^cf^	3.5 ± 0.2 ^ae^	3.0 ± 0.3 ^ae^	3.7 ± 1.1 ^ae^	4.3 ± 0.7 ^a^	3.9 ± 0.7 ^ad^	4.2 ± 0.2 ^ab^	4.3 ± 0.2 ^a^	3.5 ± 0.2 ^ae^	3.9 ± 0.8 ^ac^
Acetic acid	1.3 ± 0.5 ^a^	0.8 ± 0.3 ^a^	1.2 ± 0.4 ^a^	1.7 ± 0.6 ^a^	1.4 ± 0.8 ^a^	1.3 ± 0.1 ^a^	0.8 ± 0.1 ^a^	1.1 ± 0.4 ^a^	1.3 ± 0.6 ^a^	1.4 ± 0.8 ^a^
	**UTA11**	**UTA12**	**UTA13**	**UTA14**	**UTA15**	**UTA16**	**UTA17**	**UTA18**	**UTA19**	
Lactic acid	3.5 ± 0.5 ^ae^	3.4 ± 0.4 ^ae^	3.1 ± 0.2 ^ae^	2.3 ± 0.6 ^af^	4.1 ± 1.7 ^ab^	3.9 ± 0.9 ^ad^	3.5 ± 0.5 ^ae^	2.2 ± 1.0 ^af^	3.3 ± 0.4 ^ae^	
Acetic acid	1.1 ± 0.2^a^	1.4 ± 0.3 ^a^	1.7 ± 0.4 ^a^	1.0 ± 0.4 ^a^	1.5 ± 0.7 ^a^	1.5 ± 0.3 ^a^	2.0 ± 0.3 ^a^	0.3 ± 0.6 ^a^	1.5 ± 0.3 ^a^	
	**UTA20**	**UB5**	**UB6**	**UB7**	**UB8**	**UBC1**	**UTC6**	**UT5**	**VBC9**	
Lactic acid	3.2 ± 0.4 ^abcde^	1.8 ± 0.4 ^bf^	nd	3.1 ± 0.5 ^ae^	3.0 ± 0.8 ^ae^	1.3 ± 0.5 ^ef^	2.9 ± 0.2 ^ae^	1.4 ± 0.3 ^df^	2.9 ± 1.6 ^abe^	
Acetic acid	1.5 ± 0.3 ^a^	1.7 ± 0.4 ^a^	2.2 ± 0.6 ^a^	2.1 ± 0.4 ^a^	1.8 ± 0.6 ^a^	1.4 ± 0.3 ^a^	1.3 ± 0.5 ^a^	1.4 ± 0.3 ^a^	1.3 ± 0.1 ^a^	
**(b) Phenolic compounds**	**UTA1**	**UTA2**	**UTA3**	**UTA4**	**UTA5**	**UTA6**	**UTA7**	**UTA8**	**UTA9**	**UTA10**
Phenyllactic acid	1.3 ± 0.1 ^ad^	1.0 ± 0.1 ^dg^	1.4 ± 0.2 ^ab^	1.2 ± 0.1 ^ad^	1.2 ± 0.1 ^ad^	1.4 ± 0.1 ^ab^	0.8 ± 0.1 ^fh^	1.0 ± 0.1 ^cf^	1.4 ± 0.1 ^a^	1.3 ± 0.1 ^ac^
	**UTA11**	**UTA12**	**UTA13**	**UTA14**	**UTA15**	**UTA16**	**UTA17**	**UTA18**	**UTA19**	
	0.8 ± 0.1 ^fh^	0.7 ± 0.1 ^gh^	0.9 ± 0.1 ^fh^	0.9 ± 0.1 ^eg^	1.2 ± 0.1 ^ad^	0.8 ± 0.0 ^fh^	0.6 ± 0.1 ^h^	0.8 ± 0.1 ^fh^	1.2 ± 0.1 ^be^	
	**UTA20**	**UB5**	**UB6**	**UB7**	**UB8**	**UBC1**	**UTC6**	**UT5**	**VBC9**	
	0.9 ± 0.1 ^fh^	nd	nd	0.8 ± 0.1 ^fh^	0.8 ± 0.1 ^fh^	nd	0.8 ± 0.1 ^gh^	nd	0.6 ± 0.1 ^h^	

## Data Availability

The data used to support the findings of this study are included within article.

## Supplementary Materials

The following are available online at https://www.mdpi.com/article/10.3390/toxins13060412/s1, Table S1: Identification and quantification of the main VOC present in the CFS. Results as a percentage (%) of the VOC by dividing the area of each peak by the total area of the chromatogram peaks. Statistically significant differences for each fermentation are indicated with different letters (*p* < 0.05).
