# Supplementary material for: Bio-Preservative Potential of Microorganisms Isolated from Red Grape against Food Contaminant Fungi
Source: Toxins (Basel). 2021 Jun 10;13(6):412. doi: 10.3390/toxins13060412 (PMC8230461; doi:10.3390/toxins13060412)
Supplement: Supplementary file 1 [file toxins-13-00412-s001.zip › toxins-1228626-supplementary.pdf]

Supplementary Materials: Bio-Preservative Potential of Microorganisms Isolated from Red Grape Against Food Contaminant Fungi

Victor Dopazo, Carlos Luz, Jordi Mañes, Juan Manuel Quiles, Raquel Carbonell, Jorge Calpe and Giuseppe Meca

Table S1. Identification and quantification of the main VOC present in the CFS. Results as a percentage (%) of the VOC by dividing the area of each peak by the total area of the chromatogram peaks. Statistically significant differences for each fermentation are indicated with different letters ($p < 0.05$). nd = not detected

VOCs	Samples									
	**MRS**	**UTA1**	**UTA2**	**UTA3**	**UTA4**	**UTA5**	**UTA6**	**UTA7**	**UTA8**	**UTA9**
**ALCOHOLS**	37.8 ± 3.1^{a}	16.6 ± 1.8^{ab}	13.3 ± 2.2^{bcde}	13.0 ± 0.6^{bcde}	8.7 ± 2.1^{fghi}	12.3 ± 3.1^{cdef}	16.1 ± 0.6^{abc}	4.3 ± 0.4^{ijk}	8.4 ± 1.1^{fgh}	3.7 ± 0.1^{jkl}
Ethanol	nd	11.1 ± 0.3	8.4 ± 1.8	9.8 ± 0.1	8.7 ± 2.1	12.3 ± 3.1	12.9 ± 0.3	3.3 ± 0.3	8.4 ± 1.1	nd
2-Ethyl-1-hexanol	37.8 ± 3.1	5.4 ± 1.5	4.9 ± 0.4	3.2 ± 0.5	nd	nd	3.2 ± 0.3	1.0 ± 0.1	nd	3.7 ± 0.1
**ALDHEYDES**	27.8 ± 1.0^{bcd}	24.9 ± 5.0^{bcdefg}	15.8 ± 3.4^{hij}	19.3 ± 1.7^{efghij}	12.7 ± 1.1^{j}	17.7 ± 0.4^{fghij}	14.1 ± 2.9^{ij}	17.3 ± 1.3^{ghij}	$21.2 \pm 2.3d^{efghi}$	23.6 ± 1.2^{cdefgh}
3-Methylbutanal	nd	7.8 ± 1.8	4.0 ± 0.9	5.1 ± 1.1	nd	nd	nd	9.7 ± 0.7	4.8 ± 0.4	6.5 ± 0.0
Benzaldehyde	13.0 ± 0.8	3.6 ± 1.7	2.8 ± 0.2	4.0 ± 0.4	4.2 ± 0.8	3.8 ± 0.1	5.5 ± 0.5	1.8 ± 0.2	5.2 ± 0.4	4.7 ± 0.7
Benzeneacetaldehyde	nd	12.6 ± 0.7	7.6 ± 1.5	8.4 ± 0.1	8.5 ± 0.3	12.5 ± 0.2	8.5 ± 2.4	5.7 ± 0.4	11.3 ± 1.5	12.5 ± 0.5
3-Methylbenzaldehyde	14.8 ± 0.3	0.8 ± 0.8	1.4 ± 0.7	1.8 ± 0.2	nd	1.5 ± 0.1	nd	nd	nd	nd
PYRAZINES	nd	19.5 ± 6.4^{fg}	27.9 ± 1.2^{bcdefg}	26.9 ± 2.3^{cdefg}	32.8 ± 1.3^{abcd}	30.1 ± 2.7^{bcde}	30.9 ± 5.4^{abcd}	10.0 ± 0.7^{h}	20.2 ± 3.6^{fg}	35.2 ± 2.1^{abc}
2,5-Dimethylpyrazine	nd	11.2 ± 2.9	18.6 ± 0.4	14.2 ± 0.1	21.3 ± 0.2	14.1 ± 0.5	15.6 ± 1.4	5.4 ± 0.1	10.6 ± 1.9	22.1 ± 0.3
2-Ethyl-6-methylpyrazine	nd	nd	1.2 ± 0.2	2.3 ± 0.3	3.9 ± 0.4	3.1 ± 0.4	2.1 ± 0.8	nd	nd	3.9 ± 1.4
Trimethylpyrazine	nd	nd	0.4 ± 0.1	0.3 ± 0.1	0.8 ± 0.3	0.5 ± 0.1	0.4 ± 0.0	nd	nd	nd
2-ethyl-3-methyl-Pyrazine	nd	nd	nd	0.2 ± 0.1	nd	nd	nd	nd	nd	nd
2-ethenyl-6-methyl-Pyrazine	nd	0.6 ± 0.1	0.4 ± 0.2	0.4 ± 0.0	0.3 ± 0.1	0.7 ± 0.3	0.7 ± 0.2	nd	nd	nd
3-Ethyl-2,5-dimethylpyrazine	nd	5.7 ± 2.1	5.6 ± 0.2	6.5 ± 0.8	4.6 ± 0.2	7.6 ± 0.5	8.1 ± 2.0	3.1 ± 0.4	6.7 ± 0.6	7.2 ± 0.2
2,6-diethyl-Pyrazine	nd	0.3 ± 0.0	nd	nd	0.8 ± 0.0	nd	nd	nd	nd	nd
2-ethyl-3,5-dimethyl-Pyrazine	nd	0.1 ± 0.0	0.3 ± 0.1	0.9 ± 0.4	nd	0.8 ± 0.2	nd	nd	nd	nd
2,5-Dimethyl-3-isopentylpyra-zine	nd	1.6 ± 1.2	1.4 ± 0.0	2.1 ± 0.5	1.1 ± 0.1	3.3 ± 0.7	4.0 ± 0.9	1.6 ± 0.2	2.8 ± 1.1	2.0 ± 0.1
**OTHERS**	34.5 ± 0.7^{defgh}	39.0 ± 0.8^{bcdefg}	43.0 ± 4.3^{bcde}	40.7 ± 3.8^{bcdef}	45.8 ± 4.1^{bcd}	40.0 ± 3.2^{bcdefg}	38.9 ± 2.8^{bcdefg}	68.4 ± 1.6^{a}	50.2 ± 3.7^{b}	37.4 ± 3.2^{cdefgh}
Acetic acid	7.1 ± 0.3	27.5 ± 0.1	33.1 ± 2.1	27.5 ± 0.8	22.8 ± 1.0	23 ± 0.4	28.2 ± 0.7	65.3 ± 0.9	42.5 ± 2.8	28.0 ± 1.6
Ethyl acetate	nd	9.8 ± 0.2	5.8 ± 0.9	7.4 ± 0.9	18.2 ± 1.2	9.9 ± 1.0	5.6 ± 1.5	2.4 ± 0.7	7.7 ± 0.8	5.6 ± 1.2
Dimethyl trisulfide	nd	nd	nd	1.3 ± 0.2	1.0 ± 0.4	1.7 ± 0.5	1.3 ± 0.3	nd	nd	2.5 ± 0.4
2,3,5-trimethyl-Hexane	nd	nd	2.0 ± 0.4	1.8 ± 0.3	1.8 ± 0.4	1.7 ± 0.1	2.2 ± 0.1	0.6 ± 0.0	nd	nd
1,3-Di-tert-butylbenzene	nd	1.2 ± 0.2	0.8 ± 0.5	1.5 ± 0.2	1.3 ± 0.6	1.9 ± 0.0	1.7 ± 0.2	nd	nd	1.3 ± 0.1
2,4-Di-tert-butylphenol	27.4 ± 0.4	nd	nd	nd	nd	nd	nd	nd	nd	nd
(2,4-ditert-butylphenyl) 5-hy-droxypentanoate	nd	0.3 ± 0.3	nd	nd	nd	0.8 ± 0.1	nd	nd	nd	nd

1,5-Diphenyl-2H-1,2,4-triazoline-3-thione	nd	nd	1.3 ± 0.4	1.3 ± 1.3	0.7 ± 0.5	1.0 ± 1.1	nd	nd	nd	nd

VOCs	Samples									
	**UTA10**	**UTA11**	**UTA12**	**UTA13**	**UTA14**	**UTA15**	**UTA16**	**UTA17**	**UTA18**	**UTA19**
**ALCOHOLS**	10.4 ± 1.9 ^{defgh}	8.6 ± 1.8 ^{fgh}	6.5 ± 0.2 ^{hijk}	17.8 ± 1.2 ^{a}	9.5 ± 0.1 ^{efgh}	16.3 ± 1.8 ^{abc}	6.6 ± 0.1 ^{hijk}	7.4 ± 0.5 ^{ghij}	7.5 ± 0.1 ^{ghijk}	3.7 ± 0.4 ^{jkl}
Ethanol	7.8 ± 1.4	7.2 ± 1.8	nd	10.3 ± 0.7	nd	nd	6.6 ± 0.1	6.0 ± 0.3	7.5 ± 0.1	3.7 ± 0.4
3-methyl-1-Butanol	nd	nd	6.5 ± 0.2	7.5 ± 0.5	9.5 ± 0.1	9.7 ± 1.7	nd	nd	nd	nd
2-methyl-1-Butanol	nd	nd	nd	nd	nd	nd	nd	nd	nd	nd
2-Ethyl-1-hexanol	2.5 ± 0.6	nd	nd	nd	nd	6.6 ± 0.1	nd	1.5 ± 0.2	nd	nd
**ALDHEYDES**	20.9 ± 0.9 ^{defghi}	23 ± 2.9 ^{cdefgh}	29.8 ± 3.7 ^{bcd}	25.2 ± 2.4 ^{bcdefg}	32.7 ± 2.1 ^{b}	23.9 ± 4.0 ^{bcdefg}	41.2 ± 1.1 ^{a}	27.6 ± 1.6 ^{bcde}	25.9 ± 0.8 ^{bcdef}	30.5 ± 3.8 ^{bc}
3-Methylbutanal	8.0 ± 0.3	7.4 ± 0.8	8.5 ± 1.2	8.9 ± 1.2	11.9 ± 1.6	8.1 ± 1.7	8.6 ± 0.2	4.9 ± 0.2	9.2 ± 0.7	11.9 ± 2.4
Benzaldehyde	3.3 ± 0.3	2.8 ± 0.3	4 ± 1.3	3.3 ± 0.0	3.0 ± 0.1	3.6 ± 0.4	5.9 ± 0.2	3.7 ± 0.2	3.9 ± 0	4.6 ± 1.0
Benzeneacetaldehyde	9.7 ± 0.3	11.1 ± 1.5	14.7 ± 1.0	13 ± 1.2	17.8 ± 0.4	12.1 ± 1.9	16.6 ± 0.4	13.8 ± 1.1	12.8 ± 0.1	14 ± 0.4
3-Methylbenzaldehyde	nd	1.7 ± 0.3	2.6 ± 0.2	nd	nd	nd	5.2 ± 0.2	3.5 ± 0.1	nd	nd
5-Methyl-2-phenyl-2-hexenal	nd	nd	nd	nd	nd	nd	4.8 ± 0.1	1.8 ± 0.1	nd	nd
**PYRAZINES**	26.1 ± 1.5 ^{cdefg}	19.1 ± 1.5 ^{gh}	20.8 ± 2.7 ^{efg}	27.9 ± 4.4 ^{bcdefg}	27.7 ± 1.5 ^{bcdefg}	21.6 ± 1.8 ^{efg}	23.9 ± 0.8 ^{defg}	30.1 ± 1 ^{bcde}	34.8 ± 0.9 ^{abc}	40.3 ± 4.2 ^{a}
2,5-Dimethylpyrazine	17.4 ± 1.1	12.9 ± 0.6	14.3 ± 1.3	16.1 ± 1.8	20.8 ± 0.6	17.6 ± 0.9	8.5 ± 0.2	14.1 ± 0.5	16.5 ± 0.5	22.6 ± 2.4
2-Ethyl-6-methylpyrazine	1.9 ± 0.2	1.1 ± 0.2	nd	7.0 ± 1.0	nd	nd	nd	3.5 ± 0.1	nd	nd
3-Ethyl-2,5-dimethylpyrazine	5.0 ± 0.0	2.9 ± 0.4	4.4 ± 0.7	3.0 ± 1.0	4.7 ± 0.7	2.7 ± 0.5	6.3 ± 0.2	7.2 ± 0.2	12 ± 0.1	10.3 ± 1.2
2,6-diethyl-Pyrazine	nd	0.6 ± 0.0	nd	nd	nd	nd	nd	nd	nd	nd
2-ethyl-3,5-dimethyl-Pyrazine	nd	nd	nd	nd	nd	nd	nd	nd	nd	2.4 ± 0.4
2,6-Dimethyl-3(2-methyl-1-butyl)pyrazine	nd	nd	nd	nd	nd	nd	0.9 ± 0.0	0.6 ± 0.1	0.8 ± 0	0.7 ± 0.2
2,5-Dimethyl-3-isopentylpyrazine	1.9 ± 0.1	1.6 ± 0.3	2.2 ± 0.6	1.9 ± 0.6	2.2 ± 0.1	1.3 ± 0.4	8.3 ± 0.4	4.7 ± 0.2	5.5 ± 0.3	4.3 ± 0.1
OTHERS	42.6 ± 4.0 ^{bcde}	49.3 ± 2.9 ^{bcd}	42.9 ± 6.2 ^{bcde}	29.1 ± 2.0 ^{fgh}	30.1 ± 3.1 ^{fgh}	38.2 ± 3.3 ^{bcdefg}	28.4 ± 0.9 ^{gh}	34.9 ± 0.9 ^{defgh}	31.8 ± 0.9 ^{efgh}	25.5 ± 4.1 ^{h}
Acetic acid	28.4 ± 2.0	21.6 ± 0.7	23.3 ± 1.9	18.3 ± 0.9	20.0 ± 2.1	26.2 ± 2.1	12.9 ± 0.3	17.5 ± 0.2	21.9 ± 0.6	13.4 ± 2.3
Ethyl acetate	9.6 ± 1.0	4.9 ± 0.0	nd	3.2 ± 0.1	nd	3.9 ± 0.7	nd	3.8 ± 0.1	nd	nd
1,1-dimethyl- Cyclopropane	nd	10.3 ± 0.7	nd	nd	nd	nd	nd	nd	nd	nd
Dimethyl trisulfide	2.3 ± 0.2	5.1 ± 0.6	8.1 ± 1.7	5.6 ± 0.6	7.6 ± 0.5	6.2 ± 0.2	3.0 ± 0.0	4.7 ± 0.4	4.0 ± 0.2	4.5 ± 0.6
4-ethyl-Decane	nd	nd	nd	nd	nd	nd	nd	nd	nd	2.1 ± 0.5
Ethanone, 1-(1H-pyrrol-2-yl)-	nd	1.6 ± 0.2	2.3 ± 0.5	0.8 ± 0.1	1.0 ± 0.5	2.0 ± 0.3	3.6 ± 0.1	3.5 ± 0.2	nd	nd
3,6-dimethyl-Decane	nd	nd	nd	nd	nd	nd	nd	nd	nd	2.0 ± 0.3
2,3,5-trimethyl-Hexane	nd	1.4 ± 0.0	2.6 ± 0.6	nd	nd	nd	nd	nd	nd	nd
1,3-Di-tert-butylbenzene	nd	2.6 ± 0.1	4.7 ± 0.8	nd	nd	nd	3.9 ± 0.1	2.1 ± 0.1	3.8 ± 0.1	nd
2,4-Di-tert-butylphenol	nd	nd	nd	nd	nd	nd	5.0 ± 0.4	3.3 ± 0.0	2.0 ± 0.0	3.5 ± 0.4
(2,4-ditert-butylphenyl) 5-hydroxypentanoate	0.9 ± 0.5	0.7 ± 0.1	nd	nd	nd	nd	nd	nd	nd	nd
1,5-Diphenyl-2H-1,2,4-triazoline-3-thione	1.4 ± 0.3	1.1 ± 0.4	1.9 ± 0.5	1.2 ± 0.2	1.6 ± 0.1	nd	nd	nd	nd	nd

VOCs	Samples								
	**UTA20**	**UB5**	**UB6**	**UB7**	**UB8**	**UBC1**	**UTC6**	**UT5**	**VBC9**
**ALCOHOLS**	9.4 ± 0.9 ^{efgh}	3.6 ± 0.2 ^{kl}	4.2 ± 0.9 ^{jk}	3.2 ± 0.2 ^{kl}	11 ± 2.2 ^{fgh}	nd	13.6 ± 0.9 ^{jk}	nd	9.9 ± 1.4 ^{abcd}

Ethanol		4.1 ± 0.6	nd	nd	nd	6.0 ± 1.1	nd	8.8 ± 0.2	nd	3.4 ± 0.4
3-methyl-1-Butanol		5.2 ± 0.3	3.6 ± 0.2	nd	nd	5.0 ± 1.1	nd	4.8 ± 0.7	nd	3.9 ± 0.9
2-methyl-1-Butanol		nd	nd	nd	nd	nd	nd	nd	nd	2.7 ± 0.2
2-Ethyl-1-hexanol		nd	nd	4.2 ± 0.9	3.2 ± 0.2	nd	nd	nd	nd	nd
**ALDHEYDES**		26.1 ± 1.9^{bcdef}	26.6 ± 1.4 ^{bcdef}	31.0 ± 0.9 ^{bc}	26.5 ± 3.8 ^{bcdef}	19.4 ± 2.4 ^{defghi}	31.2 ± 2.5 ^{bc}	24.0 ± 2.1 ^{bcdefg}	24.5 ± 3.9 ^{bcdefg}	29 ± 3.2 ^{bcd}
3-Methylbutanal		5.9 ± 0.1	6.7 ± 0.4	18.5 ± 0.5	9.7 ± 0.8	nd	8.5 ± 0.7	4.2 ± 0.9	6.2 ± 1.6	4.1 ± 0.3
2-Methylbutanal		nd	nd	nd	nd	nd	nd	nd	nd	1.9 ± 0.1
Benzaldehyde		4.6 ± 0.4	2.0 ± 0.0	nd	nd	5.2 ± 0.4	nd	3.2 ± 0.0	nd	4.2 ± 0.6
Benzeneacetaldehyde		15.6 ± 1.3	8.5 ± 0.2	7.3 ± 0.3	16.8 ± 2.9	9.6 ± 0.5	9.9 ± 0.2	9.2 ± 0.2	7.6 ± 0.5	10.8 ± 0.7
3-Methylbenzaldehyde		nd	9.4 ± 0.8	5.2 ± 0.0	nd	4.7 ± 1.4	12.8 ± 1.5	4.5 ± 0.1	10.7 ± 1.8	4.6 ± 0.6
5-Methyl-2-phenyl-2-hexenal		nd	nd	nd	nd	nd	nd	2.9 ± 0.9	nd	nd
**PYRAZINES**		36.8 ± 2.8^{ab}	33.0 ± 1.6 ^{abcd}	31.5 ± 3.2 ^{abcd}	19.8 ± 1.1 ^{fg}	20.6 ± 1.1 ^{efg}	29.2 ± 5.3 ^{bcdef}	19.3 ± 1.1 ^{gh}	31.6 ± 3.3 ^{abcd}	24.9 ± 3.5 ^{defg}
2,5-Dimethylpyrazine		22.7 ± 0.6	20.2 ± 0.6	31.5 ± 3.2	16.8 ± 1.0	12.6 ± 0.6	23.7 ± 3.7	7.9 ± 0.1	17.6 ± 0.6	10.1 ± 0.9
2-Ethyl-6-methylpyrazine		nd	3.3 ± 0.4	nd	nd	nd	4.2 ± 1	nd	3.3 ± 0.8	nd
Trimethylpyrazine		nd	1.8 ± 0.1	nd	nd	nd	nd	nd	nd	nd
3-Ethyl-2,5-dimethylpyrazine		7.2 ± 1.4	2.3 ± 0.3	nd	1.8 ± 0.1	4.5 ± 0.3	nd	3.4 ± 0.5	3.5 ± 0.7	4.6 ± 0.1
2,6-diethyl-Pyrazine		nd	nd	nd	nd	nd	nd	2.2 ± 0.3	nd	nd
2-ethyl-3,5-dimethyl-Pyrazine		nd	nd	nd	nd	nd	nd	nd	nd	3.1 ± 1.6
2-Isoamyl-6-methylpyrazine		nd	1.5 ± 0.1	nd	nd	nd	nd	nd	nd	nd
2,6-Dimethyl-3(2-methyl-1-butyl)pyrazine		0.7 ± 0.2	0.8 ± 0.0	nd	nd	0.4 ± 0.0	1.3 ± 0.7	nd	1.3 ± 0.7	0.8 ± 0.1
2,5-Dimethyl-3-isopentylpyrazine		6.3 ± 0.6	nd	nd	1.2 ± 0.0	3.1 ± 0.1	nd	5.8 ± 0.3	3.5 ± 0.2	6.2 ± 0.8
2-butyl-3,5-dimethyl-Pyrazine		nd	3.2 ± 0.1	nd	nd	nd	nd	nd	2.5 ± 0.3	nd
**OTHERS**		27.8 ± 3.3^{gh}	36.8 ± 2.4 ^{cdefgh}	33.4 ± 2.9 ^{efgh}	50.5 ± 3.9 ^{b}	49 ± 5.9 ^{bc}	39.5 ± 2.6 ^{bcdefg}	43.1 ± 3.1 ^{bcde}	44 ± 3.7 ^{bcde}	36.2 ± 4.2 ^{defgh}
Acetic acid	13.2 ± 0.9	8 ± 0.4	8.9 ± 0.7	25.1 ± 0.8	21.8 ± 1.4	5.1 ± 1.0	14.3 ± 0.3	5.5 ± 0.9	11.0 ± 1.5	
Ethyl acetate	5.1 ± 1.3	nd	nd	5.4 ± 0.1	8.1 ± 1.5	nd	6.5 ± 1.4	nd	3.3 ± 0.2	
1,1-dimethyl- Cyclopropane	nd	nd	nd	12.4 ± 0.7	nd	nd	nd	nd	nd	
Dimethyl trisulfide	3.6 ± 0.9	2.6 ± 0.0	nd	7.7 ± 2.3	5.7 ± 1.5	nd	2.3 ± 0.2	nd	2.7 ± 0.2	
4-ethyl-Decane	nd	nd	nd	nd	2 ± 0.3	nd	2.3 ± 0.2	nd	2.3 ± 0.3	
Ethanone, 1-(1H-pyrrol-2-yl)-	nd	3.3 ± 0.0	nd	nd	4.6 ± 0.1	nd	3.5 ± 0.1	nd	2.5 ± 0.3	
3,6-dimethyl-Decane	nd	nd	nd	nd	1.8 ± 0.1	nd	2.6 ± 0.1	nd	1.4 ± 0.2	
5-methyl-Undecane	nd	nd	nd	nd	nd	nd	nd	nd	5.0 ± 0.4	
1,3-Di-tert-butylbenzene	5.9 ± 0.1	21.1 ± 1.9	24.5 ± 2.2	nd	1.9 ± 0	34.4 ± 1.6	7.0 ± 0.2	38.5 ± 2.9	5.1 ± 0.5	
2,4-Di-tert-butylphenol	nd	1.7 ± 0.1	nd	nd	2.0 ± 0.7	nd	2.3 ± 0.1	nd	2.7 ± 0.6	

1,5-Diphenyl-2H-1,2,4-triazoline-3-thione	nd	nd	nd	nd	nd	nd	2.4 ± 0.4	nd	nd
